# Brazilian biorepository to support genome-wide association studies of colorectal, breast, and cervical cancer

**DOI:** 10.1016/j.isci.2026.116758

**Published:** 2026-07-27

**Authors:** Lázaro Antonio Campanha Novaes, Rafaela Dias Oliveira, Isabella Lemuqui Tegami, Maria Fernanda Santiago Gonçalves, Howard Lopes Ribeiro Junior, Mariana Bisarro dos Reis, Daniel Antunes Moreno, Júlio Possati-Resende, Florinda Santos, Cláudio Hashimoto, Augusto Antoniazzi, Stefano Baraldo, Luis Romagnolo, Ricardo dos Reis, Luciane Sussuchi da Silva, Letícia Ferro Leal, Denise Peixoto Guimarães, Márcia Maria Chiquitelli Marques, Adeylson Guimarães Ribeiro, Rui Manuel Reis

**Affiliations:** 1 Molecular Oncology Research Center, Barretos Cancer Hospital, Barretos, São Paulo State, Brazil; 2 Life and Health Sciences Research Institute (ICVS), School of Medicine, University of Minho, Braga, Portugal; 3 Center for Research and Drug Development (NPDM), Federal University of Ceará, Fortaleza, Ceará, State of Ceará, Brazil; 4 Department of Prevention, Barretos Cancer Hospital, Barretos, São Paulo, Brazil; 5 Department of Clinical Oncology, Barretos Cancer Hospital, Barretos, São Paulo, Brazil; 6 Department of Endoscopy, Barretos Cancer Hospital, Barretos, São Paulo, Brazil; 7 Department of Oncogenetics, Barretos Cancer Hospital, Barretos, São Paulo, Brazil; 8 Department of Lower Surgery, Barretos Cancer Hospital, Barretos, São Paulo, Brazil; 9 Department of Gynecology Oncology, Barretos Cancer Hospital, Barretos, São Paulo, Brazil; 10 University of Health Sciences Dr Paulo Prata, FACISB, Barretos, São Paulo State, Brazil

**Keywords:** genome-wide association studies, biorepository, cancer genomics, precision medicine, genetic diversity, breast cancer, colorectal cancer, cervical cancer

## Abstract

Genome-wide association studies (GWAS) have identified numerous genetic variants associated with cancer susceptibility. However, over 90% of participants are of European ancestry, limiting the applicability to highly admixed groups such as Brazilians. To address this gap, we established a large-scale Brazilian biorepository to support cancer GWAS, replication studies, and polygenic risk score (PRS) evaluation for colorectal, breast, and cervical cancers. 4,062 participants have been enrolled, including cancer cases and cancer-free controls, yielding 39,700 blood-derived biospecimens. Genotyping was performed in 2,844 participants using the Axiom 850K PMDA array. All Brazilian states were represented, with the majority of participants from the Southeast region. Genetic ancestry inference confirmed Brazil’s highly admixed genetic background, with predominant European, followed by African, Native American, and Asian contributions. This biorepository provides a scalable resource to enable replication of genetic associations, assess the transferability of polygenic risk scores, and advance precision medicine in underrepresented populations.

## Highlights

• Large Brazilian biorepository established to support cancer GWAS in admixed populations
• Over 4,000 participants and ∼40,000 biospecimens collected nationwide
• Enables equitable evaluation of cancer polygenic risk scores across ancestries
• Mitigates gaps in cancer genomics for underrepresented Latin American populations

## Graphical abstract

**Figure:**
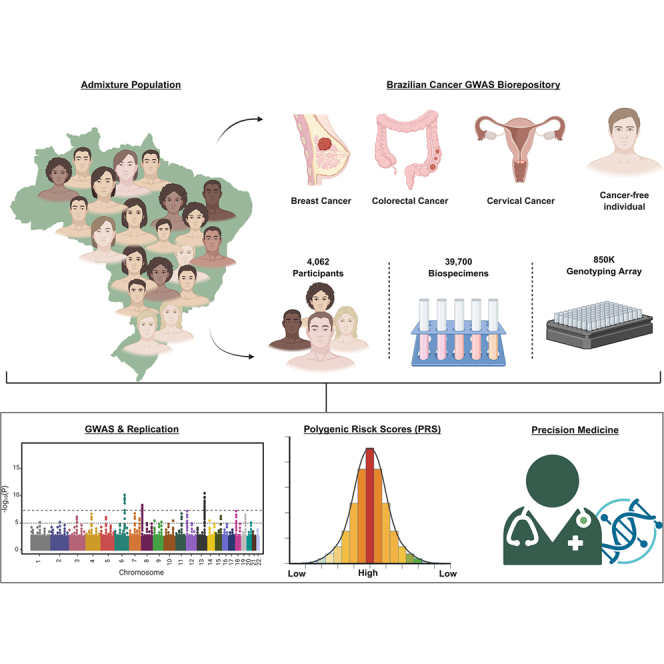

## Teaser

Public health; Cancer

## Introduction

Cancer is a major public health and economic burden in the 21st century, with nearly 20 million new cases and 9.7 million cancer-related deaths reported in 2022.^1^ According to GLOBOCAN 2022, the global cancer incidence is projected to rise by 77%, reaching 35 million new cases annually by 2050.^1^ In Brazil, data from the Instituto Nacional de Câncer (INCA) indicate a substantial national cancer burden, with high incidence of breast, colorectal, and cervical cancers, particularly among underserved populations.^2^ It is a polygenic, multifactorial disease that arises from complex interactions between genetic susceptibility, environmental and lifestyle factors, including diet, physical activity, infections, tobacco use, alcohol consumption, occupational exposures, and socioeconomic status.^3^^,^^4^ These factors contribute not only to disease onset but also to disparities in outcomes across populations.

The emergence of precision medicine has transformed the approach to cancer prevention, diagnosis, and treatment by considering individual variability in genetics, environment, and lifestyle.^5^^,^^6^ Advances in genomic technologies, particularly high-throughput sequencing and genome-wide association studies (GWAS), have enabled the identification of thousands of genetic variants associated with cancer risk across diverse populations.^7^^,^^8^^,^^9^ Among genetic markers, single-nucleotide polymorphisms (SNPs) are the most common type of variation, accounting for approximately 90% of human genetic heterozygosity.^10^^,^^11^ SNPs, due to their high frequency, stability, and low genotyping cost, are widely used in GWAS to investigate disease associations.^12^

**Figure 1:**
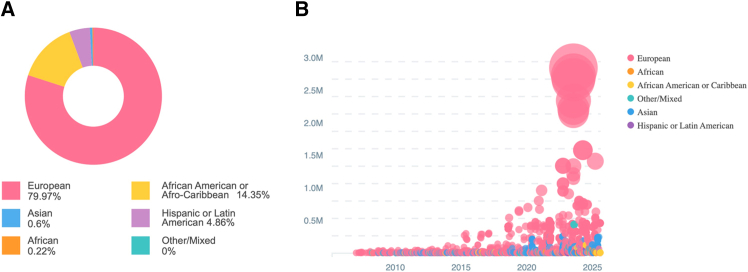
Distribution of cancer-related genome-wide association studies (GWAS) participants across ancestries (A) Cumulative number of cancer-related GWAS participants, revealing the dominance of participants of European descent (>65%). (B) Temporal trends in cancer-related GWAS participants from 2007 to 2024, highlighting the dominance of individuals of European descent, as reflected by the size of the pink bubble, with numbers increasing exponentially over time. In contrast, the representation of other minority populations remains significantly lower, with minimal growth observed in recent years (*Adapted from Mills and Rahal (2020)*.^17^ Source: https://gwasdiversitymonitor.com (Accessed on 6th August 2025).

Landmark initiatives such as the International HapMap Project, launched in 2002, enabled rapid cataloging of over 3.1 million human SNPs, providing a foundation for genomic studies of complex diseases.^13^^,^^14^ Moreover, the Wellcome Trust Case Control Consortium conducted one of the first comprehensive GWAS, leading to a surge in discoveries linking genetic variants to various diseases.^15^ Since then, GWAS have identified over 50,000 significant associations with disease risk, particularly in cancer.^16^ However, despite the volume of data, population diversity remains a significant gap in GWAS research. Over 90% of GWAS participants are of European ancestry, with admixed populations, such as Brazilians, comprising less than 2% in cancer-related studies.^17^ This underrepresentation limits the applicability of findings and contributes to disparities in precision medicine outcomes for non-European and admixed populations (Figure 1).^18^^,^^19^^,^^20^

This lack of diversity is especially concerning in countries like Brazil, where extensive ethnic and genomic admixture results from Indigenous, African, and European ancestries.^21^ Additionally, environmental exposures, dietary patterns, geographic variation, and occupational risks, particularly in middle-income countries, further influence cancer susceptibility.^22^ Such diversity highlights the importance of including these populations in genomic research to promote equity and improve the accuracy of health risk assessment predictions.

Large population-based projects like the UK Biobank, which collected genomic and health data from 500,000 British individuals, illustrate the power of population-specific biobanks in discovering new disease associations and guiding public health efforts.^23^^,^^24^ Establishing similar infrastructure in other global regions is crucial. One promising tool that relies on GWAS data is the polygenic risk score (PRS), which aggregates the effects of multiple genetic variants to estimate an individual’s risk of developing a disease. While PRSs hold great potential, their predictive accuracy depends heavily on the population in which they are designed and validated.^25^^,^^26^ Without adequate representation of diverse populations, PRSs may be less effective, or even misleading, in non-European groups.^27^

While Brazil has established significant longitudinal cohorts, such as the ELSA-Brasil (focused primarily on cardiovascular and metabolic diseases),^28^ and maintains tumor biobanks at reference centers like the Instituto do Câncer de São Paulo (ICESP), the A.C. Camargo Cancer Center, and the National Cancer Institute (INCA), these resources are generally organized around specific disease, institutions, or research projects. Consequently, there remains a scarcity of consolidated biorepositories specifically designed to support high-throughput GWAS across multiple tumor types with harmonized genomic and epidemiological data.

To address this gap, our project establishes a dedicated Brazilian biorepository to support GWAS focused on breast, colorectal, and cervical cancers. This resource prospectively aims to recruit 8,000 participants, including 2,000 cancer-free controls and 2,000 patients for each tumor type, all linked to comprehensive epidemiological questionnaires, enabling robust population-specific analyses in a highly admixed context. Rather than functioning as a *de novo* discovery GWAS, the primary objective is to enable replication of previously reported genetic associations, to evaluate and calibrate PRS transferability, and to contribute to a multicenter meta-analysis. By enhancing the representation of Brazil’s admixed population, this biorepository provides the necessary infrastructure to support equitable cancer genomics research, improving cancer risk prediction and precision prevention strategies.

## Results

### Biorepository construction

The biorepository was established within the Molecular Oncology Research Center facilities at Barretos Cancer Hospital (BCH; Hospital de Amor de Barretos), a philanthropic tertiary referral cancer center operating within the Brazilian Public Health System (SUS) and serving patients from all regions of Brazil. The initiative leverages an established institutional biobank infrastructure, operational since 2006, which currently manages more than 420,000 biospecimens from over 55,000 donors and is a member of the BCNet (Biobank and Cohort Building Network.^29^

This consolidated infrastructure provided the operational framework for standardized biospecimen collection, processing, storage, and data integration in the present study. The institutional biobank has previously supported multiple molecular and translational oncology studies and contributed to national and international collaborative initiatives, including large-scale cancer genomics efforts, such as the US-LACRN, TCGA, ICGC, and Mutographs. These existing capabilities ensured scalability, quality control (QC), and long-term sustainability of the biorepository described here.^30^^,^^31^^,^^32^

**Figure 2:**
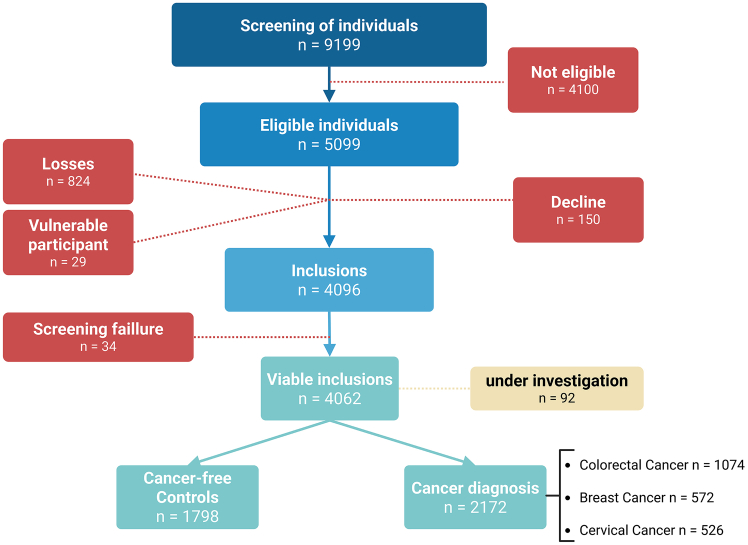
Screening-log of individuals eligible for the study and viable inclusions of cancer and cancer-free participants

**Figure 3:**
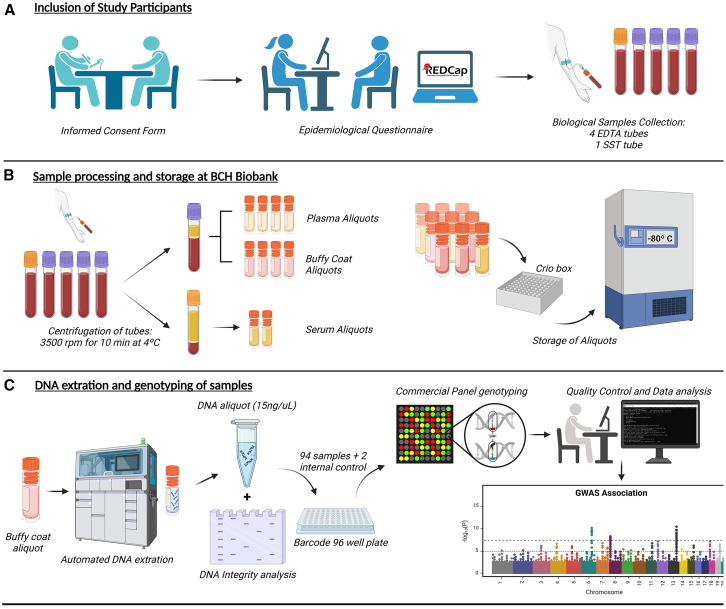
Project workflow for building the sample biorepository and database (A) Process of inclusion of participants with the completion of an epidemiological questionnaire and collection of biological material for the biorepository. (B) Processing of blood samples from participants and storage of processed buffy-coat in the Barretos Cancer Hospital Biobank (BCH Biobank). (C) Extraction of DNA from buffy-coat to perform genotyping of samples to assess risk variants for participants with cancer.

To date, 9,199 individuals have been screened for study selection on the Departments of Mastology, Gynecology, and Colorectal, as well as the Department of Prevention (Figures 2 and 3). Of these, 5,099 met eligibility criteria. However, 853 were not enrolled due to their inability to provide consent or other vulnerability criteria. Thirty-four participants were excluded after enrollment due to the subsequent identification of one of the study exclusion criteria. Ultimately, 4,062 individuals were enrolled, including 1,074 with colorectal cancer, 572 with breast cancer, 526 with cervical cancer, and 1,798 cancer-free controls. The goal is to further expand this cohort to reach 8,000 participants, comprising 2000 cancer-free controls and 2000 patients for each tumor type, thereby enhancing the statistical power for future replication analyses, fine-mapping of established loci, and exploratory gene-environment interaction studies.

**Figure 4:**
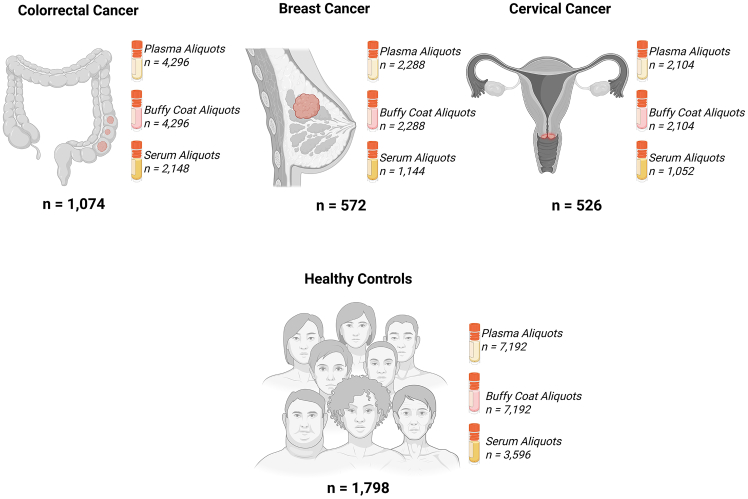
Blood-derived samples originated from all cancer types studied and cancer-free individuals

Regarding the collected samples, a total of 39,700 blood-derived biospecimens were generated, comprising 15,880 plasma, 15,880 buffy coat, and 7,940 serum aliquots. Each participant contributed up to 10 aliquots, based on the availability of sample types (Figure 4).

**Figure 5:**
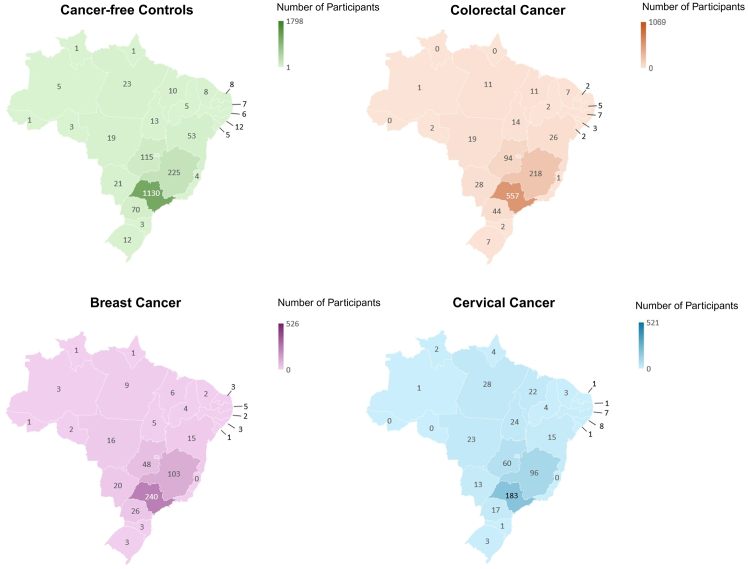
Number of inclusions by cancer type and for cancer-free individuals The maps illustrate the geographic distribution of cases and controls across Brazil. Participants are from all Brazilian regions.

A summary of the sociodemographic variables of participants is described in Table 1. The mean age of health controls was 53. For participants with colorectal cancer, the mean age was 54, and for participants with breast and cervical cancer, the mean ages were 49 and 41, respectively. Most participants self-identified as white, had completed less than secondary education, were non-smokers, reported occasional alcohol use, and had no family history of cancer. Geographically, most participants resided in Brazil’s Southeast region, although all states were represented (Figure 5).

**Table 1:** Summary of epidemiological data of participants separated by topography and the control group

Variables	Controls *n* = 1798 *n* (%)	Colorectal cancer *n* = 1074 *n* (%)	Breast cancer *n* = 572 *n* (%)	Cervical cancer *n* = 526 *n* (%)
Age				
maximum	78	83	79	70
minimum	20	19	23	20
mean	53	54	49	41
Sex^a^				
female	1175 (65.4)	486 (45.3)	572 (100)	526 (100)
male	623 (34.6)	588 (54.7)	–	–
Self-declared skin color^b^				
black	182 (10.1)	74 (6.9)	34 (5.9)	53 (10.1)
brown/mixed	631 (35.1)	356 (33)	219 (38.2)	271 (51.5)
indigenous	–	2 (0.2)	–	–
white	948 (53)	623 (58)	312 (54.5)	195 (37.1)
yellow	15 (0.8)	18 (1.7)	7 (1.2)	7 (1.3)
unknown	22 (1.2)	1 (0.1)	–	–
Education				
illiterate	11 (0.6)	14 (1.3)	9 (1.6)	13 (2.5)
elementary school	589 (33)	529 (49.3)	220 (38.5)	160 (30.4)
high school	634 (35)	324 (30.2)	186 (32.5)	238 (45.2)
higher school	341 (19)	153 (14.2)	112 (19.6)	78 (15)
complete postgraduate	215 (12)	54 (5)	45 (7.9)	37 (7)
undefined	8 (0.4)	–	–	–
Religion				
catholic	720 (40)	570 (53.1)	208 (36.4)	211 (40.1)
protestant	334 (18.6)	211 (19.6)	109 (19.1)	137 (26)
spiritist	57 (3.2)	43 (4)	21 (3.7)	14 (2.7)
other	30 (1.4)	26 (2.1)	11 (1.7)	19 (3.6)
none	70 (3.9)	38 (3.5)	4 (0.7)	20 (3.8)
unknown	587 (32.6)	184 (17.1)	219 (38.3)	125 (24)
Smoking status				
never	1211 (67.4)	591 (55)	377 (66)	356 (67.7)
current	202 (11.2)	115 (10.7)	65 (11.4)	81 (15.4)
past	360 (20)	364 (33.9)	117 (20.4)	85 (16.2)
unknown	25 (1.4)	4 (0.4)	13(2.2)	4(0.7)
Alcohol consumption				
never	551 (30.6)	280 (26.1)	220 (38.5)	154 (29.3)
current	1027 (57.1)	486 (45.3)	222 (39)	243 (46.2)
past	196 (10.9)	304 (28.3)	118 (20.6)	125 (23.8)
unknown	24 (1.3)	4 (0.4)	16(2.8)	4(0.7)
Sun exposure^c^				
no exposure	463 (25.8)	381 (35.5)	173 (30.2)	148 (28.1)
6-7 days/week	724 (40.3)	432 (40.2)	198 (34.6)	190 (36.1)
3-5 days/week	290 (16.1)	183 (17)	95 (16.6)	116 (22.1)
1-2 days/week	161 (9)	42 (3.9)	49 (8.5)	42 (8)
every fifteen days	13 (0.7)	2 (0.2)	12 (2.1)	3 (0.6)
once a month	13 (0.7)	3 (0.3)	6 (1)	3 (0.6)
unknown	134(7.5)	31 (2.9)	39(6.8)	24(4.5)
Physical activity				
never	652 (36.3)	464 (43.2)	243 (42.4)	255 (48.5)
current	735 (40.9)	300 (27.9)	189 (33)	148 (28.1)
past	386 (21.5)	306 (28.5)	125 (22)	119 (22.6)
unknown	25 (1.4)	4 (0.3)	15(2.6)	4(0.7)
Family history of cancer^d^				
yes	551 (30.6)	245 (22.8)	136 (23.8)	144 (27.4)
no	1216 (67.6)	823 (76.6)	388 (67.8)	374 (71.1)
unknown	31 (1.7)	6(0.6)	2 (0.3)	8 (1.5)

a Sex assigned at birth.

b Self-reported skin color according to the Brazilian Institute of Geography and Statistics (IBGE).

c Sun exposure of less than 1 h/day is considered as no exposure.

d First or second degree relatives with cancer.

### Genotyping samples

Out of 4,062 participants with stored biospecimens, 2,844 DNA samples have been submitted for genotyping using the Axiom PMDA platform, and the remaining cases are ongoing. Among these, 980 are from colorectal cancer patients, 457 from breast cancer, 239 from cervical cancer, and 1,168 from controls. Across all groups, over 96.9% of evaluated SNPs met the clustering criteria. The failure rates caused by low Dish QC (DQC <0.82) or genotyping call rates (QC < 97%) were low and are explained in the following section.

Based on QC of Axiom analysis suite, for samples from individuals with no previous history of cancer (cancer-free control group), a total of 846,519 SNPs were genotyped, and 97.49% of the SNPs evaluated were clustered within the quality parameters established. One sample failed genotyping, and four samples did not pass QC due to QC values below 97%.

Among the colorectal cancer samples, 980 passed QC, with a total of 845,236 SNPs being evaluated; of these, 97.34% (*n* = 953) met the genotyping quality cluster criteria. Among the failed samples, three samples had a DQC value < 0.82, and 24 samples presented a QC value < 97%. For breast cancer samples, 457 passed QC, with 842,210 SNPs genotyped. Of these, 97% (*n* = 444) are clustered within the defined quality parameters. Thirteen samples failed QC due to QC values below 97%. Regarding cervical cancer samples, 239 passed to QC, with 846,577 SNPs genotyped, and 97.5% (*n* = 235) within the quality thresholds. Four samples failed QC due to QC values below 97%.

### Ancestry composition of the cancer and non-cancer individuals

**Figure 6:**
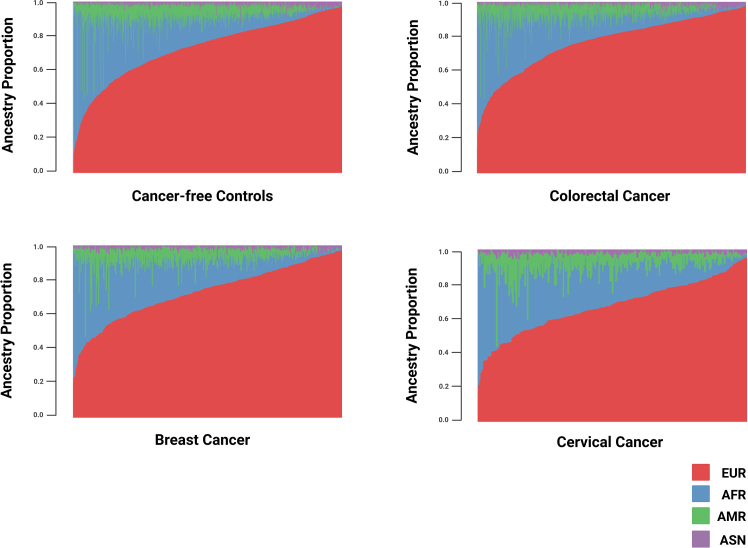
Genetic ancestry composition of the Brazilian cohorts estimated using ADMIXTURE v1.3.0 Graphical representation of individual ancestry proportions for each cohort, namely cancer-free controls, colorectal cancer, breast cancer, and cervical cancer. Each vertical bar represents one individual and their ancestry proportion. European (EUR) is red, African (AFR) is blue, Native American (AMR) is green, and Asian (ASN) is purple.

The Brazilian population is highly admixed, and this diversity is reflected in the ancestry profiles of the studied cohorts (Table 2 and Figure 6). After sample and variant QC of the genotyping data using PLINK software, 3082 samples were approved (911 colorectal cancer, 431 breast cancer, 225 cervical cancer, and 1515 controls) and analyzed for evaluation of ancestry components (Table S2). The analyses revealed that EUR ancestry showed a significantly higher mean proportion in colorectal cancer patients (0.7673 ± 0.1621) compared with controls (0.7292 ± 0.1843; *p* < 0.001), whereas no significant difference was observed in breast cancer patients (0.7289 ± 0.1660; *p* = 0.333). Cervical cancer patients presented a significantly lower EUR proportion (0.6654 ± 0.1718; *p* < 0.001). African (AFR) ancestry displayed an opposite pattern, with significantly lower proportions in colorectal cancer (0.1569 ± 0.1484; *p* < 0.001) compared with controls (0.1944 ± 0.1759) and significantly higher proportions in cervical cancer patients (0.2278 ± 0.1618; *p* < 0.001). No significant differences were detected in AFR proportions for breast cancer (*p* = 0.695) (Table 2).

**Table 2:** Mean and standard deviation (SD) of each ancestry proportion across groups

	Controls	Colorectal cancer	Breast cancer	Cervical cancer
EUR	0.73 ± 0.18	0.77 ± 0.16	0.73 ± 0.17	0.66 ± 0.17
AFR	0.19 ± 0.18	0.16 ± 0.15	0.19 ± 0.15	0.23 ± 0.16
AMR	0.06 ± 0.06	0.06 ± 0.05	0.07 ± 0.05	0.08 ± 0.06
ASN	0.01 ± 0.01	0.02 ± 0.01	0.02 ± 0.01	0.02 ± 0.01

Ancestry proportions reported in mean and standard deviation within each cohort: breast cancer, cervical cancer, colorectal cancer, and cancer-free controls. European ancestry (EUR), African ancestry (AFR), Native American ancestry (AMR), and Asian ancestry (ASN).

Native American (AMR) ancestry was slightly higher in breast cancer patients (0.0686 ± 0.0538) compared with controls (0.0641 ± 0.0571; *p* = 0.031) and significantly higher in cervical cancer patients (0.0845 ± 0.0601; *p* < 0.001), whereas no significant difference was found for colorectal cancer (*p* = 0.172). Asian (ASN) ancestry proportions were significantly higher across all cancer cohorts compared with controls (colorectal: 0.0151 ± 0.0142, *p* < 0.001; breast: 0.0161 ± 0.0128, *p* < 0.001; cervical: 0.0221 ± 0.0132, *p* < 0.001) (Table 2). These findings highlight the heterogeneous ancestral composition of the Brazilian cohorts and suggest that differences in ancestry proportions may contribute to the observed variability across cancer types.

## Discussion

This study describes the establishment of a nationwide biorepository integrating colorectal, breast, and cervical cancer cases with cancer-free controls from the highly admixed Brazilian population. To date, the initiative has recruited over 4,000 participants, generated nearly 40,000 high-quality biospecimens, and successfully genotyped more than 2,800 samples under stringent quality-control criteria. Beyond sample collection, the primary contribution of this effort lies in the creation of a scalable genomic resource designed to support replication studies, ancestry-aware analyses, and evaluation of PRS transferability in a population historically underrepresented in large-scale cancer genomics research.

The Brazilian population exhibits varying degrees of European, African, and Amerindian ancestry throughout its territory, regions, and states.^21^^,^^33^^,^^34^ This offers a unique opportunity to explore how genetic variation affects the risk of certain types of cancer in highly mixed populations.^21^^,^^33^^,^^34^^,^^35^^,^^36^^,^^37^^,^^38^^,^^39^^,^^40^ This biorepository reflects the genetic heterogeneity across Brazil’s regions, with participants recruited from all 26 states and the Federal District. The significant variation in African and Native American ancestry proportions observed between our cancer and control groups demonstrates the complex population substructure that often confounds risk association studies. These findings reinforce that relying solely on Eurocentric genomic data would likely introduce stratification bias, highlighting the critical need for this biorepository to validate global hits and calibrate polygenic risk scores for Latin American populations. Furthermore, based on the Brazilian ancestral configuration, it may be possible to identify more than one model for predicting polygenic risk for tumors in Brazil, due to the distribution of ancestral proportions across the country’s regions.

The ancestry analysis results for both cancer and cancer-free individuals revealed the highly admixed genetic background of the Brazilian population. Overall, European ancestry was the most predominant, followed by African, Native American, and, to a lesser extent, Asian ancestry. These findings are consistent with recent studies of Brazilian cohorts,^34^^,^^41^^,^^42^ which also reported a complex, highly admixed genetic ancestry profile. Therefore, it is necessary to account for population substructure in genetic analyses and to highlight the importance of ancestry-adjusted models for cancer risk prediction in Brazil.

In addition to biological samples, this study has created a comprehensive and detailed epidemiological dataset. While most large-scale GWAS rely on minimal phenotype data, usually limited to age, sex, and case-control status, this study incorporated extensive lifestyle, environmental exposure, clinical characteristics, and family history. Although the current sample size limits the statistical power for definitive interaction testing, this rich dataset facilitates exploratory analyses of gene-environment interactions and generates hypotheses regarding how local behavioral factors may modify genetic susceptibility. This level of integration can foster precision oncology approaches in Latin America.

The initial genotyping results demonstrate that high-throughput SNP array technologies are compatible with biospecimens collected under real-world, biobank conditions. Genotyping success rates exceeded 96% across all cancer types and controls, with many SNPs passing stringent clustering and QC thresholds, thereby validating the integrity of the sample processing pipeline. Although a small number of samples failed to meet quality metrics, repeat genotyping is ongoing, underscoring the feasibility of recovering and analyzing longitudinal samples from institutional biobanks.

In the future, this biorepository will support two complementary avenues of investigation: (1) replication and validation of previously reported cancer-associated SNPs in a highly admixed population, and (2) evaluation of population-specific patterns of genetic risk through fine-mapping and assessment of PRS transferability. While this resource may contribute to hypothesis generation, its primary value lies in strengthening the generalizability, calibration, and equity of cancer genomics research in Latin America. If clinically validated, such biomarkers could support early cancer detection, personalized risk stratification, and more efficient resource allocation in public health systems.

Furthermore, the scientific value of this resource is already shown in a recent study that used a subset of colorectal cancer and cancer-free participants to replicate European-derived GWAS risk variants, thereby identifying specific ancestry-linked risk loci in the Brazilian population.^34^^,^^41^

When comparing this resource to other initiatives in South America, it is important to distinguish between broad population-based cohorts and disease-specific genomic biobanks. Large-scale longitudinal studies such as the ELSA, and EPIGEN-Brazil cohorts, PoblAr in Argentina, and the MAUCO cohort in Chile have provided invaluable data on ancestry and chronic non-communicable diseases, mainly cardiovascular and metabolic disorders.^28^^,^^43^^,^^44^^,^^45^ However, there is a relative scarcity of biobanks specifically designed for cancer GWAS in the region. Unlike initiatives focused solely on tumor tissue profiling or general epidemiology, this biorepository integrates high-density germline genotyping with epidemiological data specifically for colorectal, breast, and cervical cancers. Additionally, by recruiting from a tertiary center in the Brazilian interior rather than exclusively from major capital cities, our cohort captures a unique profile of the country’s continental admixture, providing a distinct dataset for cross-validation of findings from other Latin American consortia.

Despite its strengths, this study has important limitations that should be acknowledged. Although the biorepository provides broad national coverage, with participants recruited from all Brazilian states, recruitment from a single tertiary referral center may underrepresent certain historically underserved regions, particularly in the North and Northeast of the country. As such, this resource should be viewed as foundational and scalable infrastructure rather than a fully representative national cohort. To address this limitation, an ongoing Ministry of Health-funded initiative (PRONON) is expanding the biobank network by establishing additional regional sites across Brazil, including centers in the Western Amazon, the far North, and the Northeast. This stepwise expansion is expected to enhance geographic, socioeconomic, and ancestry diversity, strengthening the resource’s representativeness and long-term sustainability. Another important limitation concerns statistical power. While the current sample size is well suited for replicating previously reported genetic associations, evaluating and calibrating PRS transferability, and participating in multicenter meta-analyses, it is not optimized for *de novo* discovery of low-effect variants typical of large-scale GWAS. Accordingly, findings derived from this resource should be interpreted within this intended scope.

In conclusion, this study exemplifies a highly relevant and innovative translational research initiative. By integrating epidemiological and genomic data from an admixed population, it contributes to the global understanding of cancer genetics while addressing the pressing need for population-specific insights. The resulting biorepository not only expands knowledge of tumorigenesis in diverse groups but also holds promise for real-world applications in cancer prevention, screening, and personalized care across Brazil and similarly underrepresented genomic populations worldwide.

### Limitations of the study

Although participants were recruited nationwide, enrollment at a single tertiary referral center may limit geographic and socioeconomic representativeness. The current sample size is adequate for replication analyses and assessment of PRS transferability, but is underpowered for *de novo* discovery of low-effect GWAS variants. In addition, epidemiological data were self-reported and may be subject to recall bias. Ongoing cohort expansion and multicenter recruitment are expected to mitigate these limitations.

## Resource availability

### Lead contact

For further information please contact Rui Manuel Reis (ruireis.hcb@gmail.com).

### Materials availability

This study did not generate new unique materials.

### Data and code availability

• Data: Individual-level SNP-array genomic data generated in this study have been deposited in the European Genome-phenome Archive under accession number EGAS00001008408. The study accession/metadata are publicly available through the EGA portal. Because the datasets contain sensitive human genomic data, the underlying individual-level data are available only through EGA controlled access, subject to approval by the relevant Data Access Committee and applicable consent, ethics, institutional, and data protection requirements.
• Code: The code used in this study has been deposited in a public GitHub repository (https://github.com/Rafaeladoliveira/BrazilianBiorepository_project). This repository includes scripts for ancestry analysis and data QC steps. If additional information is needed, it can be requested from the lead contact.
• Other items: Additional information required from this article will be available upon request to the lead contact.

## Acknowledgments

This research was funded by the 10.13039/501100006506Brazilian Ministry of Health, supported by PRONON/MS: (NUP-25000.023997.2018/34) entitled “Identificação de biomarcadores para screening e detecção precoce de tumores no contexto do Sistema Único de Saúde (SUS)”; and (10.13039/100027697NUP: 25000.192948/2019-21) entitled “Criação da rede de Biobancos do Hospital de Câncer de Barretos”. H.L.R.J., A.G.R., and D.A.M. were recipients from PRONON/MS (NUP25000.023997.2018/34) of postdoctoral fellowships; M.B.R. is a recipient of a post-doc fellowship grant # 2021/13861-0, 10.13039/501100001807São Paulo Research Foundation (FAPESP). L.A.C.N., I.L.T., and M.F.G. received grants from PRONON/MS (NUP25000.023997.2018/34) to work as study coordinators and biologists; R.D.O. received a PhD fellowship from 10.13039/501100001871Portuguese FCT (2023.00584.BD). R.M.R., L.F.L., and H.L.R.J. are recipients of CNPq productivity fellowships. The authors appreciate initial discussions with Ana Carolina de Carvalho and Alexandre C. Pereira.

## Author contributions

R.M.R., and L.F.L. designed the study. L.A.C.N., M.F.S.G., I.L.T., H.L.R.J., M.B.R., D.A.M., J.P.-R., F.S., C.H., A.A., S.B., L.R., R.R., R.P.G. and M.M.C.M. collected data. L.A.C.N., R.D.O., L.S.S., and A.G.R. analyzed the data. L.A.C.N., R.D.O., I.L.T., and M.F.S.G. and R.M.R. drafted the article. All authors contributed to data interpretation, review, and approved the final version of the manuscript.

## Declaration of interests

The authors declare no competing interests.

## STAR★Methods

### Key resources table

**Table:** 

REAGENT or RESOURCE	SOURCE	IDENTIFIER
**Critical commercial assays**		

Qiasyphony DSP DNA Midi Kit (96)	Qiagen	https://www.qiagen.com/br - Catalog Number: 931255
Axiom™ Precision Medicine Diversity Array Kit, 96-format	Thermo Fisher Scientific	https://www.thermofisher.com/br - Catalog Number: 951962

**Deposited data**		

Raw data	This paper	https://ega-archive.org/ as [EGAS00001008408]

**Software and algorithms**		

Axiom™ Analysis Suite v4.0	Thermo Fisher Scientific	https://www.thermofisher.com/br
pLink v1.90b06.21	COG-Genomics	https://www.cog-genomics.org/plink/
Admixture v1.3	https://doi.org/10.1101/gr.094052.109	https://dalexander.github.io/admixture/

**Other**		

Scripts and code	This paper	https://github.com/Rafaeladoliveira/BrazilianBiorepository_project

### Experimental model and study participant details

Adult participants (≥18 years) with confirmed diagnoses of colorectal, breast, or cervical cancer, as well as cancer-free controls, were prospectively recruited at Barretos Cancer Hospital (BCH). This study was conducted between January/2020 and December/2024 (Figure 2). Cases were ascertained from the Departments of Colorectal, Mastology, and Gynecology for confirmation of a primary diagnosis of colorectal, breast, or cervical cancer, respectively. All participants provided written informed consent under a protocol approved by the Institutional Review Board of Barretos Cancer Hospital (IRB protocol number: 16940619.5.0000.5437).

The cancer-free control group was recruited from two complementary sources within the same institutional setting: (i) individuals undergoing routine cancer screening at the hospital’s prevention unit who presented with negative findings (e.g., routine mammography, colonoscopy, or pap smear), and (ii) unrelated companions of patients. This strategy ensures that controls share the same catchment area, environmental exposures, and socioeconomic background as the cases, but without a personal history of cancer.

### Method details

#### Study setting

The study was conducted at Barretos Cancer Hospital, a tertiary national cancer referral center. As a philanthropic institution within the Brazilian Public Health System (SUS), BCH provides comprehensive, free-of-charge cancer care to all patients. BCH receives patients from all 26 Brazilian states and the Federal District, with over 1 million attendances annually (https://hospitaldeamor.com.br). Patients are typically referred from primary and secondary care units across the country through the national regulation system (CROSS). This broad national coverage ensures that the study cohort captures the population’s genomic and socioeconomic diversity.

#### Sample/data collection and inclusion criteria

Eligible participants were individuals aged 18 years or older at the time of diagnosis. For the cancer groups, inclusion required a confirmed diagnosis of colorectal, breast, or cervical cancer, and treatment-naïve. Blood samples were collected from both cancer and cancer-free participants and stored at the institutional Biobank.^29^ Individuals with a previous history of other cancers or a known hereditary cancer syndrome were excluded.

Data collection included the application of a lifestyle/epidemiological questionnaire, which lasted approximately 15 to 20 minutes (Epidemiological Survey S1). Subsequently, peripheral blood samples were collected for processing and storage in the institutional biobank. All processes were carried out by trained professionals, certified to collect both epidemiological data and biological samples from participants (Figure 6A).

#### Epidemiological and clinical questionnaire

Data from prospectively recruited cancer patients and control individuals were collected and recorded using a database built on the Research Electronic Data Capture (REDCap) platform.^46^ Modeled on the ‘Oral Health Survey: Basic Methods’ (5^th^ edition) published by the World Health Organization (WHO),^2^ we designed a questionnaire including the following key categories: (a) demographic information, including age, sex, ethnic origin, place of birth and residence, educational attainment, financial status, and religion; (b) tobacco use history, covering frequency and intensity of consumption; (c) alcohol consumption history, detailing frequency and intensity; (d) lifestyle factors, such as physical activity levels; (e) anthropometric data, including height and weight (at diagnosis for cancer patients); (f) sun exposure, specifying frequency, preferred times of exposure, and use of protective measures; (g) dietary habits prior to diagnosis; (h) endocrine factors and reproductive history, specifically for female participants; (i) medical history related to comorbidities other than the diagnosed cancer, such as hypertension, diabetes, and hypercholesterolemia; (j) family history of cancer; and (k) occupational history, including job type, work frequency, and exposure to occupational hazards (Epidemiological Survey S1). As part of the questionnaire process, all participants were asked to self-report as belonging to one of the ethnic groups used by the Brazilian Institute of Geography and Statistics (IBGE) in the national census (White, Black, Yellow, Pardo, translated as Brown/Mixed, and Indigenous).^47^ The database will also include clinical, histopathological, and molecular information of the patients. The topics covered are (i) clinical diagnosis, which includes detailed information on tumor type, histological classification, localization, presence and location of metastases, and disease stage; (ii) surgical interventions, specifying whether surgery was performed and, if so, the details of the procedure; (iii) molecular diagnosis, including relevant genetic and biomarker data; (iv) clinical treatment history, detailing the type of therapeutic interventions (e.g., surgery, chemotherapy, radiotherapy, immunotherapy), tumor response to treatment, and the number of treatment lines administered to the patient; and (v) overall survival data, which includes the patient’s current status (alive, in remission, or deceased) and, where applicable, the cause of death.

Clinical research coordinators were responsible for recruiting and consenting the patients, and provided instructions for completing the forms and collected the questionnaires. These data will be crucial for supporting further adjustment of GWAS results for environmental and demographic covariates.

#### Blood collection and DNA isolation

From each participant, blood samples were collected using One BD Vacutainer® Serum Separator Tubes II Advance Tube (SST) and four BD Vacutainer® (K2EDTA) (Becton, Dickinson and Company, Franklin Lakes, NJ, USA), which were processed within 4 hours. The SST and K2EDTA tubes were manually processed by centrifugation at 3500 rpm for 10 minutes at 4°C to separate components (Figure 6B). Following this initial centrifugation, the buffy coat was aliquoted, and the plasma fraction was transferred to a new tube for a second centrifugation at 12000 rpm at 10°C for 4 minutes. Subsequently, up to two aliquots of serum, four aliquots of plasma, and four aliquots of buffy coat (1 mL each) were prepared and stored at -80°C until use at Barretos Cancer Hospital biobank (Figure 6B).^29^

For DNA isolation, one fresh-frozen buffy coat aliquot from each participant was used for genomic DNA (gDNA) extraction using the QIAGEN QIAsymphony® SP automated system (QIAGEN, Hombrechtikon, Switzerland). Briefly, the buffy coat aliquots were thawed in a water bath at 37°C for 5 minutes and treated with 3 μL of RNase A (Thermo Fisher Scientific Inc., Waltham, MA, USA). Subsequent gDNA extraction was performed using the QIAsymphony DSP DNA Midi Kit (QIAGEN GmbH, Hilden, Germany), following the manufacturer’s instructions with minor in-house modifications, including the use of 400 μL of buffy coat as the starting sample volume and an elution volume of up to 100 μL. (Figure 6C). The isolated gDNA samples were assessed for purity using the A260/A280 and A260/A230 ratios with the NanoDrop One Spectrophotometer (Thermo Fisher Scientific Inc., Waltham, MA, USA). Quantification was performed using the Invitrogen™ Qubit 2 Fluorometer (Invitrogen™, Thermo Fisher Scientific Inc., Waltham, MA, USA) and the Broad Range dsDNA Assays Kit (Thermo Fisher Scientific Inc., Waltham, MA, USA). The isolated gDNA was aliquoted and diluted to a final concentration of 1000 ng in 60 μL. Samples that did not reach the minimum concentration of 16.6 ng/μL were excluded. A minimum total of 750 ng in 60 μL is required for SNPs genotyping protocol.

A 3 μL fraction of each sample was analyzed for gDNA integrity via electrophoresis on a 1.5% agarose gel, using GelRed® Nucleic Acid Gel Stain 10,000X Water (Biotium Glowing Products for Science™, Fremont, CA, USA) and the 1 Kb Plus DNA Ladder (Invitrogen™, Thermo Fisher Scientific Inc., Waltham, MA, USA) as molecular weight markers. Finally, the samples were transferred to 96-well plates, properly sealed, and stored at -30°C until they were sent for genotyping by the Axiom Precision Medicine Diversity Array assay (Axiom PMDA, Thermo Fisher Scientific). In each 96-well plate, a repetition of 10 cases was included for further reproducibility purposes (Figure 6C).

#### SNP genotyping

SNP genotyping of cancer and non-cancer participants was performed using the commercial 850k Axiom^TM^ Precision Medicine Diversity Array (Axiom PMDA, Thermo Fisher Scientific). The Axiom PMDA array comprises over 850,000 pan-ethnic biomarkers selected from the 1000Genomes Project phase III and variants included in databases (ClinVar, NHGRI-GWAS, CPIC, PharmaGKB and PharmaADME). This panel covers the five major ancestral groups and includes cancer-related markers identified in recent GWAS publications catalogued by the European Bioinformatics Institute, as well as unpublished cancer-associated SNPs. The genotyping methodology is based on the principle of allele-specific PCR and utilizes the reagents of the Axiom 2.0 Plus kits and the Affymetrix GeneTitan Multichannel Instrument. Genotyping data, generated as.CEL files, were subjected to quality control using the Applied Biosystems™ Axiom™ Analysis Suite software (ThermoFisher Scientific).

Individual-level SNP-array genomic data generated in this study have been deposited in the European Genome-phenome Archive under accession number EGAS00001008408. The study accession/metadata are publicly available through the EGA portal. Because the datasets contain sensitive human genomic data, the underlying individual-level data are available only through EGA controlled access, subject to approval by the relevant Data Access Committee and applicable consent, ethics, institutional, and data protection requirements.

### Quantification and statistical analysis

#### Quality control of genotype data

Samples with Plate Quality Control (DQC) scores and quality control (QC) call rates below 0.82 and 97%, respectively, were excluded. Data quality control was performed using PLINK software (version 1.9) in a Linux environment, including custom R scripts. Samples and variants were excluded based on the following criteria: a missingness rate greater than 2%, minor allele frequency (MAF) less than 5%, significant deviation from Hardy–Weinberg Equilibrium (HWE) expectations (*p*-value < 1 × 10^−6^ for controls and *p* < 1 × 10^−10^ for cases), and relatedness up to the second degree (first- and second-degree kinship). After quality control, the data will be submitted to GWAS association analyses and subsequent association tests.

#### Genetic ancestry evaluation

The genetic ancestry of cancer and non-cancer participants was estimated using ADMIXTURE (v1.3.0),^48^ a model-based software that adopts a maximum likelihood approach to infer individual ancestries from multilocus SNP genotype data. Supervised analysis was conducted using reference samples from the 1000 Genomes Project, after excluding the admixed American individuals, in combination with 44 Native American samples from the Human Genome Diversity Project. The number of postulated ancestral populations was set as K=4, corresponding to European (EUR), African (AFR), Asian (ASN), and Native American (AMR) groups. Each participant was classified based on the proportion of genetic ancestry attributed to these four continental groups. The graphical analysis for admixture proportions was performed in the R language.

## Supplemental information

Document S1. Data S1/Methods S1

Table S1. Individual ancestry components in Brazilian samples S2, related to Figure 5
