# Supplementary material for: Brazilian biorepository to support genome-wide association studies of colorectal, breast, and cervical cancer
Source: iScience. 2026 Jul 27;29(8):116758. doi: 10.1016/j.isci.2026.116758 (PMC13429900; doi:10.1016/j.isci.2026.116758)
Supplement: Document S1. Data S1/Methods S1 [file mmc1.pdf]

iScience, Volume *29*

Supplemental information

Brazilian biorepository to support genome-wide association studies of colorectal, breast, and cervical cancer

Lázaro Antonio Campanha Novaes, Rafaela Dias Oliveira, Isabella Lemuqui Tegami, Maria Fernanda Santiago Gonçalves, Howard Lopes Ribeiro Junior, Mariana Bisarro dos Reis, Daniel Antunes Moreno, Júlio Possati-Resende, Florinda Santos, Cláudio Hashimoto, Augusto Antoniazzi, Stefano Baraldo, Luis Romagnolo, Ricardo dos Reis, Luciane Sussuchi da Silva, Letícia Ferro Leal, Denise Peixoto Guimarães, Márcia Maria Chiquitelli Marques, Adeylson Guimarães Ribeiro, and Rui Manuel Reis

Data S1/Methods S1: Epidemiological questionnaire applied to participants of the biorepository.

Epidemiological Survey	
**Item**	**Options**
**Screening and Eligibility Form**	
Have you had another type of cancer? (Excluding non-melanoma skin)	Yes / No / Ignored
If you have a history of previous cancer, describe	Text field
Hereditary cancer syndrome?	Yes / No / Ignored
**Participant Data**	
Questionnaire application date	Date field
Initials	Text field
Gender	Female / Male
Date of birth	Date field
**Sociodemographic Data**	
What is your education level?	Undefined / Illiterate / Incomplete elementary / Complete elementary / Incomplete high / Complete high / Incomplete higher education / Complete higher education / Incomplete postgraduate / Complete postgraduate / Ignored
What is your self-declared color/race? (As considered by the participant)	Undefined / White / Black / Yellow (Asian) / Brown / Indigenous / Cafuzo (Mixed Indigenous and African descent) / Ignored
What is your marital status?	Single / Married / Divorced / Widowed / Other / Ignored
Type of cancer treated	Colorectal Cancer / Breast Cancer / Cervical Cancer / Lung Cancer
What is your household income in minimum wages (MW)?	Up to 1 MW / More than 1 and up to 3 MW / More than 3 and up to 5 MW / More than 5 and up to 7 MW / More than 7 MW / Ignored
Do you have a religion?	Yes / No
Which religion?	Catholic / Protestant/Evangelical / Jewish / Spiritist / Buddhist / Other / Ignored
If other religion, describe	Text field
**Residence**	
What is your state of residence?	Text field
What is your municipality of residence?	Text field
Do you live in a rural or urban area?	Urban area / Rural area / Ignored
Length of residence in the municipality	Up to 1 year / More than 1 year / Ignored
What is your state of birth?	Text field
What is your municipality of birth?	Text field
Are you from a rural or urban origin?	Urban area / Rural area / Ignored
**Lifestyle Smoking**	
Do you smoke cigarettes?	Current smoker / Former smoker / Never smoked / Ignored
If former smoker, how long ago did you quit?	Time in years
If former smoker, at what age did you quit?	Age in years

What type of cigarette do you smoke/smoked?	Paper cigarette / Straw cigarette / Both (paper and straw) / Other / Ignored
If other type of cigarette, please specify	Text field
Have you ever smoked cigarettes on most days for at least one year?	Yes / No
On average, how many paper cigarettes do you smoke/smoked per day?	Number field
For how many years have you smoked paper cigarettes?	Number field
On average, how many straw cigarettes do you smoke/smoked per day?	Number field
For how many years have you smoked straw cigarettes?	Number field
**Alcohol Consumption**	
Do you consume or have you consumed alcoholic beverages?	Yes / Consumed in the past / No / Ignored
If consumed in the past, how long ago did you stop?	Time in years
How often do/did you consume beverages containing alcohol?	Once a month or less / Two to four times a month / Two to three times a week / Four or more times a week / Ignored
When you drink/drank, how many drinks containing alcohol do/did you consume on a typical day?	One or two / Three or four / Five or six / Seven to nine / Ten or more / Ignored
How often do/did you consume six or more drinks on a single occasion?	Never / Less than once a month / At least once a month / At least once a week / Daily or almost daily / Ignored
For how long have you consumed or did you consume alcohol?	Time in years
**Physical Activity**	
Do you practice or have you practiced physical exercise or sports?	No, never practiced / Yes, practiced in the past / Yes, currently practicing / Ignored
If practiced in the past, how long ago did you stop?	Time in years
How many days per week do you usually practice or used to practice physical exercise or sports?	Number field
On a day you practice/practiced physical exercise or sports, how long does/did the activity last?	Up to 30 minutes / More than 30 minutes up to 1 hour / More than 1 hour up to 2 hours / More than 2 hours / Ignored
For how long have you practiced or did you practice physical activity or sports?	Time in years
**Sun Exposure**	
How often do you expose yourself to the sun?	Six to seven days a week / Three to five days a week / One to two days a week / Every two weeks / Once a month / Do not expose / Ignored
What is your exposure time to the sun throughout the day?	Up to 1 hour / More than 1 hour up to 2 hours / More than 2 hours up to 4 hours / More than 4 hours up to 6 hours / More than 6 hours / Ignored
What is your sun exposure time of day?	Until 10 am / Between 10 am and 3 pm / After 3 pm / All morning / All afternoon / All day
What physical means do you use to protect yourself from the sun?	Hat/cap / Umbrella / Shirt / Sunglasses / Other / None
If other physical means, specify	Text field

Do you use sunscreen?	Always / Sometimes / Never / Ignored
How long have you been using sunscreen?	Time in years
Throughout your life, have you ever had any episodes of sunburn?	Yes / No / Ignored
**Diet (Food Intake Before Diagnosis)**	
Frequency of red meat consumption	Never / Less than once a month / One to three times a month / One or two times a week / Three or four times a week / Five or six times a week / Every day / Ignored
Frequency of poultry consumption	Never / Less than once a month / One to three times a month / One or two times a week / Three or four times a week / Five or six times a week / Every day / Ignored
Frequency of processed meat consumption	Never / Less than once a month / One to three times a month / One or two times a week / Three or four times a week / Five or six times a week / Every day / Ignored
Frequency of fresh fish consumption	Never / Less than once a month / One to three times a month / One or two times a week / Three or four times a week / Five or six times a week / Every day / Ignored
Frequency of processed fish consumption	Never / Less than once a month / One to three times a month / One or two times a week / Three or four times a week / Five or six times a week / Every day / Ignored
Frequency of fresh fruits consumption	Never / Less than once a month / One to three times a month / One or two times a week / Three or four times a week / Five or six times a week / Every day / Ignored
Frequency of raw or cooked vegetables consumption	Never / Less than once a month / One to three times a month / One or two times a week / Three or four times a week / Five or six times a week / Every day / Ignored
Frequency of dairy product consumption	Never / Less than once a month / One to three times a month / One or two times a week / Three or four times a week / Five or six times a week / Every day / Ignored
Frequency of cookies, chocolates, cakes, and sweets consumption	Never / Less than once a month / One to three times a month / One or two times a week / Three or four times a week / Five or six times a week / Every day / Ignored
Frequency of soda consumption	Never / Less than once a month / One to three times a month / One or two times a week / Three or four times a week / Five or six times a week / Every day / Ignored
**Endocrine Factors / Reproductive History (Section Exclusive for Female Participants)**	
Age of menarche (first menstruation)	Age in years
Age when sexual life began	Age in years
Have you ever been pregnant?	Yes / No / Ignored
Age at first pregnancy	Age in years

Number of deliveries	Number field
Did you breastfeed your children?	Yes / No / Ignored
Have you ever had an abortion?	Yes / No / Ignored
Number of abortions	Number field
Have you entered menopause?	Yes / No / Ignored
Age at menopause	Age in years
Have you ever used or are you using hormone replacement therapy?	Never / Regular use / Used before / Ignored
Duration of hormone therapy use	Time in years
Time since hormone therapy was stopped	Time in years
Have you ever used or are you using contraceptives?	Never / Regular use / Used before / Ignored
Duration of contraceptive use	Time in years
Time since contraceptive use was stopped	Time in years
Do you use condoms in sexual relations?	Always / Sometimes / Never / Ignored
How many sexual partners have you had in your life?	None / One / Two to three / Four to five / Six to seven / Eight or more / Ignored
How many sexual partners have you had in the past year?	None / One / Two / Three or more / Ignored
Have you received the HPV vaccine?	Yes / No / Ignored
Do you have regular mammogram exams?	Yes, regularly / Sometimes / Never / Ignored
How many years ago was your last mammogram?	Time in years
What was the time interval between the last two mammograms?	Time in years
Do you have regular Pap smear exams?	Yes, regularly / Sometimes / Never / Ignored
How many years ago was your last Pap smear?	Time in years
What was the time interval between the last two Pap smears?	Time in years
**Comorbidities (Consider Before Diagnosis)**	
Do you have or have you had any of the following comorbidities?	None / Tuberculosis / Diabetes / Heart disease / Liver disease / Pancreatitis / Coagulopathy / Hypertension / COPD / Asthma / Allergies / Crohn's disease / Ulcerative colitis / Chronic kidney disease / Kidney stones / Colorectal polyps / Cholangitis / GERD / Genetic hereditary disorder / Pulmonary fibrosis / Pneumonia / Emphysema / Chronic bronchitis / Myocardial infarction / Stroke / High cholesterol / Hypothyroidism / Hyperthyroidism / Thrombosis / Other / Ignored
If genetic hereditary disorder, specify	Text field
If other comorbidity, specify	Text field
Have you had any prior (non-oncological) surgery?	Yes / No / Ignored
If prior surgery, describe	Text field
Do you have a family history of cancer?	Yes / No / Ignored
Number of family members with cancer	Number field
Family member relationship	Text field
Family member cancer type	Text field
Family member age at diagnosis	Number field

Body Characteristics (Consider Weight Before Diagnosis)	
What is your weight?	Weight in kg
What is your height?	Height in cm
**Occupation**	
What is your occupation?	Text field
Occupational category	Unemployed / Administrative / Trade / Services / Industry / Civil construction / Health / Education / Domestic / Agriculture / Other (specify) / Ignored
How long have you been in this occupation?	Time in years
What is the occupation you held for the longest period?	Text field
How long have you held this occupation?	Time in years
Have you been exposed to any chemicals or substances at work?	Yes / No / Ignored
Type of activity with exposure to listed substances	Pesticides / Solvents / Disinfectants / Ionizing radiation / X-ray equipment / Smoke/motor fumes / Fuel vapors / Metal dust / Asbestos / Formaldehyde / Paints/dyes / Cement/plaster / Other / Ignored
If other substances, specify	Text field
